# Trimethylamine *n*-Oxide (TMAO) Modulates the Expression of Cardiovascular Disease-Related microRNAs and Their Targets

**DOI:** 10.3390/ijms222011145

**Published:** 2021-10-15

**Authors:** Laura Díez-Ricote, Paloma Ruiz-Valderrey, Víctor Micó, Ruth Blanco-Rojo, João Tomé-Carneiro, Alberto Dávalos, José M. Ordovás, Lidia Daimiel

**Affiliations:** 1 Nutritional Control of the Epigenome Group, Precision Nutrition and Obesity Program, IMDEA Food, UAM + CSIC, 28049 Madrid, Spain; laura.diez@imdea.org (L.D.-R.); paloma.ruiz@imdea.org (P.R.-V.); victor.mico@imdea.org (V.M.); ruth.blanco@imdea.org (R.B.-R.); jordovas@tufts.edu (J.M.O.); 2 Research and Development Department, Biosearch Life, 18004 Granada, Spain; 3 Bioactive Food Ingredients Group, Precision Nutrition and Cardiometabolic Health Program, IMDEA Food, UAM + CSIC, 28049 Madrid, Spain; joao.estevao@imdea.org; 4 Epigenetics of Lipid Metabolism Group, Precision Nutrition and Cardiometabolic Health Program, IMDEA Food, UAM + CSIC, 28049 Madrid, Spain; alberto.davalos@imdea.org; 5 Nutrition and Genomics Laboratory, JM_USDA Human Nutrition Research Center on Aging, Tufts University, Boston, MA 02111, USA

**Keywords:** TMAO, cardiovascular disease, nutrition, miRNAs, epigenetics, target genes, atherosclerosis

## Abstract

Diet is a well-known risk factor of cardiovascular diseases (CVDs). Some microRNAs (miRNAs) have been described to regulate molecular pathways related to CVDs. Diet can modulate miRNAs and their target genes. Choline, betaine, and l-carnitine, nutrients found in animal products, are metabolized into trimethylamine *n*-oxide (TMAO), which has been associated with CVD risk. The aim of this study was to investigate TMAO regulation of CVD-related miRNAs and their target genes in cellular models of liver and macrophages. We treated HEPG-2, THP-1, mouse liver organoids, and primary human macrophages with 6 µM TMAO at different timepoints (4, 8, and 24 h for HEPG-2 and mouse liver organoids, 12 and 24 h for THP-1, and 12 h for primary human macrophages) and analyzed the expression of a selected panel of CVD-related miRNAs and their target genes and proteins by real-time PCR and Western blot, respectively. HEPG-2 cells were transfected with anti-miR-30c and syn-miR-30c. TMAO increased the expression of miR-21-5p and miR-30c-5p. *PER2*, a target gene of both, decreased its expression with TMAO in HEPG-2 and mice liver organoids but increased its mRNA expression with syn-miR-30c. We concluded that TMAO modulates the expression of miRNAs related to CVDs, and that such modulation affects their target genes.

## 1. Introduction

Cardiovascular diseases (CVDs) are the first cause of death amongst developed countries. In 2017, 17.8 million people died of cardiovascular events, representing a 21.1% increase from 2007 [1]. According to the WHO, in 2019, there were 17.9 million deaths worldwide from CVD, which represents 32% of global deaths (https://www.who.int/news-room/fact-sheets/detail/cardiovascular-diseases-(cvds) (accessed on 18 August 2021). In the US, according to the Centers for Control Disease and Prevention, one in four deaths is caused by CVD, and about 647,000 people die every year (https://www.cdc.gov/nchs/fastats/heart-disease.htm (accessed on 18 August 2021)). In Europe, 42% of total deaths are caused by a cardiovascular event, constituting the leading cause of death, as well as a decline in quality of life and disability [2]. This accounts for an annual cost of approximately 200 billion EUR, which represents 54% of total healthcare investment and leads to a 24% loss in productivity [2,3]. Atherosclerosis, i.e., the plaque containing fatty acids, oxidized cholesterol, and calcium that builds within the arterial walls, is the primary cause of CVDs [4], and oxidative stress and inflammation, promoted by cholesterol-loaded macrophages infiltrated in the artery wall, are crucial to the development of atherosclerosis.

Nutrition has a significant role in health and the development of noncommunicable diseases [5]. It has been reported that 45% of cardiometabolic deaths are associated with poor nutrition [5]. In this regard, a high consumption of saturated animal fat and red meat has been linked to CVDs [6], although its role in CVD progression is not yet accurately defined. Recent meta-analyses did not find a strong association between red meat and processed meat intake and CVDs [7,8]. Dietary patterns with lower red meat intake did not show significant reductions in cardiovascular event occurrence. Likewise, a reduction of 2–3 red meat servings per week was not significantly associated with a reduction in cardiovascular risk. It should be considered that these meta-analyses are based on observational studies, in which dietary records are not always accurate [7,8]. In addition, the effect of one diet component may differ across subjects, depending on their dietary pattern [7,8].

Choline, betaine (a metabolite from choline), and l-carnitine consumption have also been associated with atherosclerosis progression and CVD development [2]. Choline is mainly found in eggs, liver, and pork meat. It is a semi-essential nutrient belonging to the vitamin B group, and it is involved in lipid and amino-acid metabolism; it is also the precursor of the acetylcholine neurotransmitter, and it is a structural component of the cell membrane [9]. l-Carnitine is mainly found in red meat, and its primary function is to transport long-chain fatty acids from the cytoplasm to the mitochondria, playing an important role in fatty-acid oxidation [2]. Both micronutrients are metabolized through the same pathway, as they have a trimethylamine structure. First, the intestinal microbiota metabolizes choline, betaine, and l-carnitine to trimethylamine (TMA). TMA is a gas that is then absorbed and easily transported to the liver, where it is metabolized to trimethylamine-*n*-oxide (TMAO) by the flavin-containing mono-oxygenase (FMO) enzyme [2].

TMAO has been previously reported to be a marker for CVDs, and it has been suggested that high plasma TMAO levels could help predict the risk of suffering certain cardiovascular adverse events when other traditional disease biomarkers, such as plasma lipids, are within normal ranges [10]. Studies in mice and humans treated with broad-spectrum antibiotics revealed that the microbiota plays an essential role in choline metabolism and, thus, in determining TMAO levels [9,10,11]. In this regard, it has been reported that TMAO levels do not increase under antibiotic treatment, due to antibiotic-mediated dysbiosis. However, after the restoration of the microbiota, choline consumption has been found to increase plasma TMAO levels. In addition, studies performed in vegetarian subjects showed that plasma TMAO levels did not increase as much as in omnivore subjects after choline consumption, suggesting that a meat-rich diet promotes a specific type of microbiota that, in turn, contributes to higher TMAO levels [3].

TMAO could also promote atherosclerosis progression as it promotes cholesterol accumulation in macrophages by inducing proatherogenic receptors CD36 and SR-A1. Changes in reverse cholesterol transport receptors ABCA1 and ABCG5 have also been reported [2,3]. However, the molecular mechanisms via which TMAO induces atheroma plaque formation are still unknown. Therefore, the effects of dietary choline, l-carnitine, and betaine consumption and its main metabolite, TMAO, are not fully understood at a molecular level.

MiRNAs are short noncoding RNA molecules that regulate gene expression by targeting mRNAs, and they play a pivotal role in fine-tuning the expression levels of their target genes in response to a stimulus. Several miRNAs have been associated with CVDs and related diseases, such as type 2 diabetes (T2D) or dyslipidemia [12], as well as with the modulation of lipid and glucose metabolism [13]. For instance, miR-21-5p [14,15] and miR-34a-5p [16] have been associated with inflammation and atherosclerosis, and miR-30c-5p has also been linked to hyperlipidemia and type 2 diabetes [17,18,19]. Other miRNAs have been reported to modulate cholesterol metabolism, such as miR-27a-3p [4], miR-181b-5p [20], miR-122-5p [21], and miR-223-3p [22], along with miR-33a-5p, miR-33b-5p [23,24,25], and miR-145-5p, which regulate reverse cholesterol transport [26]. The miR-17-92 cluster has been extensively studied due to its implication in cancerous processes, while it is also involved in endothelial function [27,28,29]. Other miRNAs have been linked to CVD progression and complications, as well as inflammation, such as miR-124-3p, miR-128-3p [30,31], miR-155-5p, miR-31-5p [32,33], miR-133a-3p, and miR-133b-3p [34,35], which are considered CVD biomarkers. Lastly, miR-132-3p has been linked to cardiac failure [36], and miR-129-5p has been shown to improve cardiac function [37]. Moreover, nutrients can modulate the expression of miRNAs at the cellular level, and the nutritional modulation of miRNAs has an impact on cellular physiology [38,39,40]. In this sense, investigating the potential impact of TMAO levels on miRNAs could help define the mechanisms underlying the association between TMAO levels and atherosclerosis and CVDs.

Therefore, this study was aimed at evaluating the effect of TMAO on the expression of miRNAs involved in lipid metabolism and related to CVDs and their targets in two relevant cellular models involved in TMAO metabolism (i.e., macrophages and hepatocytes). We found that TMAO upregulates miR-30c-5p and miR-21-5p, which modulate lipid metabolism and inflammation. We also demonstrate that TMAO downregulates *PER2*, a target gene of both miRNAs, which is involved in circadian rhythms regulation and its disruption has been linked to CVD [41]. Therefore, our study suggests that TMAO could promote inflammation and atherosclerosis through the modulation of related miRNAs and their target gene, *PER2*.

## 2. Results

### 2.1. Effect of TMAO on the Expression of CVD-Related miRNAs in HEPG-2 and THP-1 Cells

First, we performed TMAO dose–response and time–response experiments in HEPG-2 and THP-1 cells (Figures S1 and S2). For that purpose, we treated HEPG-2 and THP-1 cells with 6, 10, and 50 µM TMAO for 2, 4, 8, and 12 h and with 6 and 10 µM for 24, 48, and 72 h, respectively. We measured the expression of *ABCA1, CYP7A1*, *CD36*, and *SR-IA*, four well-known TMAO-responsive genes, to assess the TMAO effect (Figure S1). Koeth et al. studied the effect of TMAO in different mice tissues and reported that TMAO altered the expression of *Abca1* and *Cyp7a1* in liver [2] and *Cd36* and *Sr-a1* in harvested mice macrophages [3]. The lowest assayed dose of TMAO (6 µM) increased *ABCA1* expression at 2, 4, 12, and 24 h and *CYP7A1* expression at 2, 4, and 8 h in HEPG-2 cells (Figure S1a). Longer exposure times with 6 and 10 µM did not increase the expression of these genes in HEPG-2 cells. Therefore, we selected 4, 8, and 24 h as treatment timepoints for subsequent analysis of the effect of TMAO on the expression of the selected miRNAs. TMAO (6 µM) also increased the expression of *CD36* at 4, 8, 12, 24, and 72 h, and of *SR-A1* at 8, 12, 24, and 72 h in THP-1 cells (Figure S1b). Given that the effect of TMAO on the responsive genes in THP-1 was longer than in HEPG-2, we selected longer exposure timepoints for this cell line, 12 and 24 h. Higher doses did not increase the effect of 6 µM of TMAO on these genes. Thus, for subsequent experiments, we selected 6 µM as the TMAO dosage and incubation timepoints of 4, 8, and 24 h for HEPG-2 and of 12 and 24 h for THP-1.

We then analyzed the effect of TMAO (6 µM) on the expression of the selected panel of 22 miRNAs related to CVDs and lipid metabolism (Table S1). For this purpose, we treated HEPG-2 cells with 6 µM TMAO for 4, 8, and 24 h and THP-1 cells with 6 and 10 µM for 12 and 24 h, and we compared the expression of the selected miRNAs with the untreated control cells at the same timepoints. We measured the expression levels of miRNAs by RT-qPCR and detected 11 miRNAs in HEPG-2 cells and THP-1 cells.

In HEPG-2, miR-122-3p, miR128-3p, miR-133a-3p, miR-21-5p, miR-27a-3p, miR-30c-5p, and miR-92a-3p were significantly altered at one timepoint compared to their corresponding untreated cells at the same timepoint. (Table S2).

In THP-1 cells, miR-129-5p, miR-181b-5p, miR-21-5p, and miR-30c-5p were significantly altered by TMAO at one timepoint compared to their corresponding untreated cells at the same timepoint (Table S2).

Among the responsive miRNAs, miR-21-5p and miR-30c-5p were significantly altered by TMAO in both HEPG-2 and THP-1 cells at one timepoint (FDR-adjusted *p*-value < 0.05) (Table S2). miR-30c-5p was significantly upregulated by TMAO after 4 h of exposure in HEPG-2 cells, and after 24 h of exposure in THP-1 cells. miR-21-5p was significantly downregulated after 24 h of exposure in HEPG-2 cells (fold-change: 0.51; *p*-value: 0.03) and significantly upregulated after 12 h of exposure in THP-1 cells (fold-change: 1.24; *p*-value: 0.01). We previously showed that these miRNAs are modulated by dietary compounds such as fatty acids [8], beer [42], and extra virgin olive oil [40], and they also play a fundamental role in lipid metabolism, inflammation, and endothelial function [9,10,11,12,13,43].

**Figure 1:**
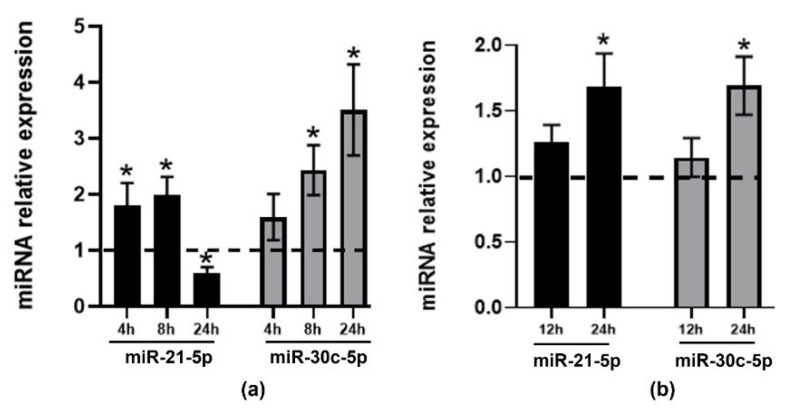
TMAO effect on the expression of miR-21-5p and miR-30c-5p in HEPG-2 and THP-1 cells. HEPG-2 (**a**) and THP-1 (**b**) cells were treated with 6 µM TMAO for 4, 8, and 24 h (HEPG-2) and 12 h and 24 h (THP-1). miR-21-5p (black bars) and miR-30c-5p (light grey bars) levels were measured by RT-qPCR. miRNA relative expression levels were calculated using the 2^−ΔΔCT^ method comparing treated cells with each corresponding untreated control sample at each timepoint. The mean ± SEM of three independent experiments is shown. Dashed lines represent the no-change threshold. * *p* < 0.05 for the comparison of each timepoint with the corresponding untreated timepoint using a Student’s *t*-test.

We then validated the observed changes in miR-30c-5p and miR-21-5p after exposure to TMAO at the defined timepoint in subsequent experimental replicates (Figure 1). In HEPG-2 cells (Figure 1a), miR-30c-5p was significantly upregulated at 8 h and 24 h. miR-21-5p expression was significantly increased in HEPG-2 at 4h and 8h and restored to control levels after 24 h. In contrast, in THP-1 cells, a significant increase in its expression was only seen after 24 h of TMAO treatment (Figure 1b).

### 2.2. Validation of Changes in miRNAs Expression in Murine Hepatic Organoids and in Human Primary Macrophages

**Figure 2:**
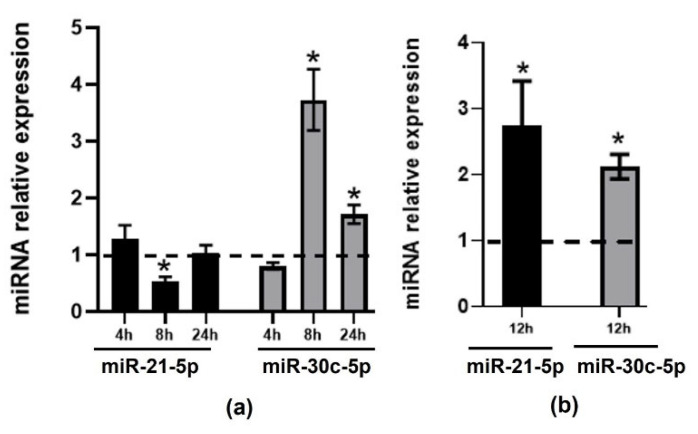
TMAO effect on the expression of miR-21-5p and miR-30c-5p in mouse liver organoids and human primary macrophages. Mouse liver organoids (**a**) and human primary macrophages (**b**) were treated with 6 µM TMAO for 4, 8, and 24 h (liver organoids) and for 12 h (human primary macrophages). miR-21-5p (black bars) and miR-30c-5p (light-gray bars) levels were measured by RT-qPCR. miRNA relative expression levels were calculated using the 2^−ΔΔCT^ method, comparing treated cells with each corresponding untreated control sample at each timepoint. The mean ± SEM of three independent experiments is shown. Dashed lines represent the no-change threshold. * *p* < 0.05 for the comparison of each timepoint with the corresponding untreated timepoint using a Student’s *t*-test.

Next, we aimed to validate results in other appropriate, non-immortalized cellular models. For this purpose, we incubated mouse liver organoids and human primary macrophages with 6 µM TMAO for 4, 8, and 24 h and for 12 h, respectively (Figure 2). Like in HEPG-2 cells, we also found that TMAO reduced the expression of miR-21 and increased the expression of miR-30c-5p in mouse liver organoids (Figure 2a). However, some differences in the impact of TMAO on human HEPG-2 cells and mouse liver organoids were observed. We found that miR-21-5p was upregulated at 8 h in HEPG-2 (Figure 1a) cells but it was downregulated at this timepoint in mouse liver organoids (Figure 2a). Our results suggest that downregulation of miR-21-5p is earlier in mouse liver organoids (8 h) than in HEPG-2 cells (24 h).

TMAO increased the expression of miR-21-5p and miR-30c-5p in human primary macrophages, validating our results in THP-1 cells.

### 2.3. Effect of TMAO on the Expression of miRNAs Target Genes and Proteins in HEPG-2 and THP-1

According to DIANA miRPath analysis, miR-30c-5p and miR-21-5p share the pathways involved in p53 signaling, cell cycle, and synthesis of fatty acids (Figure S2a). We then searched for their predicted and validated targets. Using miRWalk, we found 610 genes that were experimentally validated or predicted targets of miR-30c-5p or miR-21-5p (Tables S3 and S4). Fifteen genes were targets of both miRNAs (Figure S2b and Table S3). We carried out a functional enrichment of genes targeted by both miRNAs (*n* = 15 genes) to identify the biological processes and pathways in which those genes are involved (Table S3). We found that most target genes were involved in the regulation of metabolic processes and gene expression (Table S3). We selected *PER2*, a central regulator of the circadian system. The circadian system has a well-known role in the regulation of lipid homeostasis [14], and alterations of this system have been reported to have an impact on CVDs and atherosclerosis [15,16,17]. We aimed to study if TMAO affected *PER2* expression and if such modulation was mediated by miRNAs.

**Figure 3:**
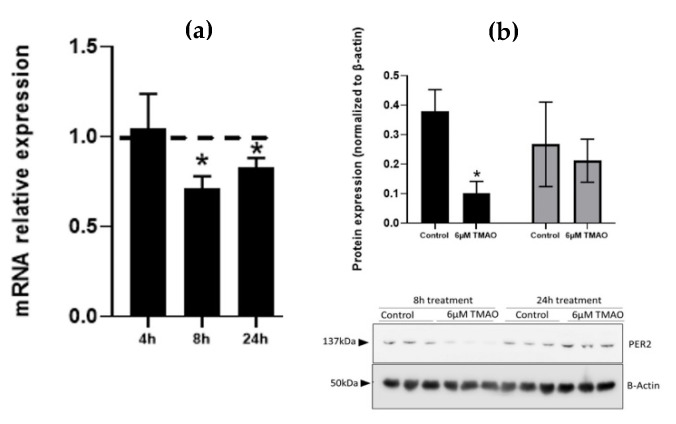
Effect of TMAO on PER2 gene and protein expression levels. Cells were treated with 6 µM TMAO for 8 h (black bars) and 24 h (gray bars), and gene (**a**) and protein (**b**) expression levels of PER2 were measured in HEPG-2 cells. Gene expression was measured by RT-qPCR and calculated using the 2^−ΔΔCT^ relative expression method, comparing treated cells with each corresponding untreated control sample at each timepoint. Protein levels were measured by Western blot. Band density was measured and normalized with β-actin. The mean ± SEM of three independent experiments is shown. Dashed lines represent the no-change threshold. * *p* < 0.05 for the comparison of each timepoint with the corresponding untreated timepoint using a Student’s *t*-test.

We measured *PER2* expression levels after exposure of HEPG-2 to 6 µM TMAO. We observed a significant decrease in *PER2* gene expression after 8 h (0.71, *p*-value = 0.002) and 24 h (0.83, *p*-value = 0.01) TMAO treatment in HEPG-2 cells (Figure 3a). PER2 protein expression was also significantly decreased after 8 h of treatment in HEPG-2 (0.1, *p*-value = 0.008) (Figure 3b). We found a similar decrease in PER2 protein expression levels in mice liver organoids (data not shown). These results are in accordance with the upregulated expression of miR-30c-5p and miR-21-5p at 8 h, in response to TMAO.

### 2.4. Effect of Anti-miRNA-30c and Syn-miRNA-30c with TMAO on the Expression of miR-30c-5p Target Genes and Protein Expression in HEPG-2

To confirm a potential interaction between miR-30c-5p and *PER2*, HEPG-2 cells were transfected with 50 nM anti-miRNA-30c or 5 nM syn-miRNA-30c and then treated with 6 µM TMAO for 4, 8, and 24 h.

**Figure 4:**
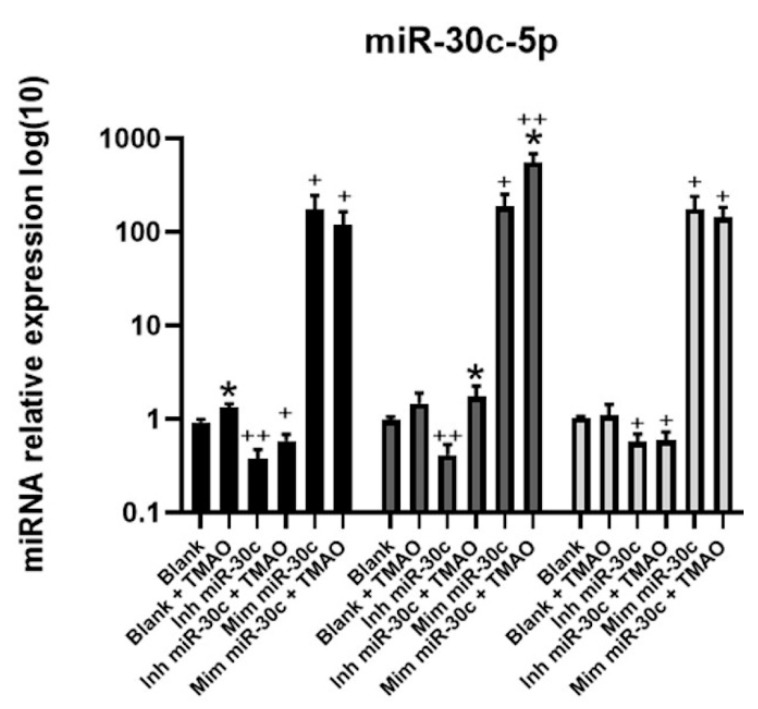
Effect of miR-30c inhibition or overexpression on TMAO-mediated regulation of miR-30c-5p in HEPG-2 cells. Cells were transfected with 50 nM anti-miRNA-30c or 5 nM syn-miRNA-30c and incubated with 6 µM TMAO for 4 (black bars), 8 (dark-gray bars), and 24 h (light-gray bars). The expression of miR-30c-5p, was measured by RT-qPCR. mRNA levels were calculated using the 2^−ΔΔCT^ relative method, comparing with mock-transfected cells (blank). The mean ± SEM of three independent experiments is shown. * *p* < 0.05 comparing each untreated condition with its corresponding TMAO-treated condition using a Student’s *t*-test; + *p* < 0.05, ++ *p* < 0.001 comparing anti-miR-30c- and syn-miR-30c-transfected samples with their corresponding non-transfected samples using a Student’s *t*-test.

To confirm the transfection, miR-30c-5p levels were measured by RT-qPCR (Figure 4). We observed that HEPG-2 transfected cells with anti-miRNA-30c showed reduced miR-30c-5p expression compared with non-transfected cells. Cells transfected with syn-miRNA-30c showed a remarkable increase in miR-30c-5p. Interestingly, cells transfected with anti-miRNA-30c and treated with TMAO showed an increase in miR-30c expression, compared to nontreated HEPG-2, mainly after 8 h, indicating that TMAO specifically induces miR-30c-5p expression, even in the presence of an inhibitor. Furthermore, HEPG-2 treated with TMAO and transfected syn-miRNA-30c showed a significant increase in miR-30c-5p at 8 h, compared to syn-miR-30c-transfected cells without TMAO treatment.

**Figure 5:**
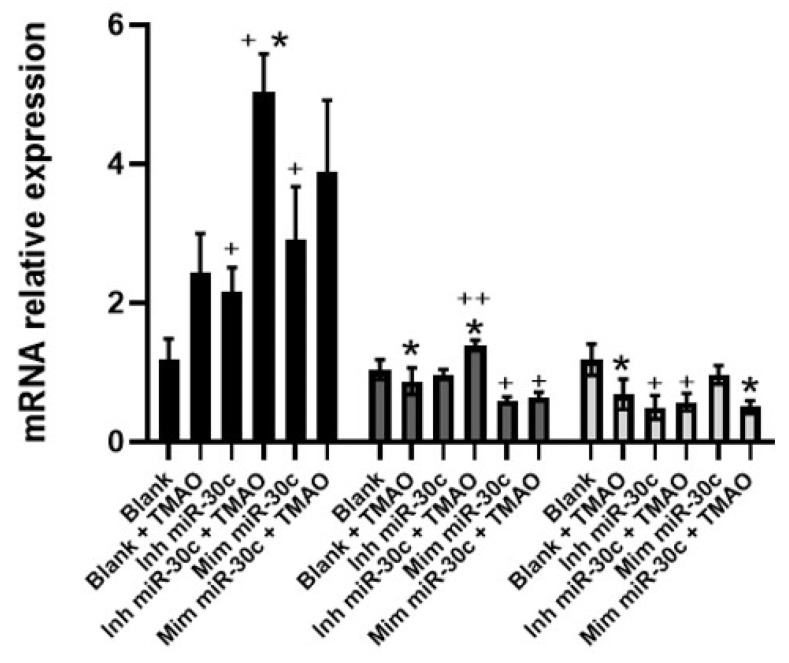
Effect of miR-30c inhibition or overexpression on TMAO-mediated regulation of *PER2* in HEPG-2 cells. Cells were transfected with 50 nM anti-miRNA-30c or 5 nM syn-miRNA-30c and incubated with 6 µM TMAO for 4 (black barks), 8 (dark-gray bars), and 24 h (light-gray bars). The expression of *PER2* was measured by RT-qPCR. mRNA levels were calculated using the 2^−ΔΔCT^ method. The mean ± SEM of three independent experiments is shown. * *p* < 0.05, comparing each untreated condition with its corresponding TMAO-treated condition using a Student’s *t*-test; + *p* < 0.05, ++ *p* < 0.001 comparing anti-miR-30c- and syn-miR-30c-transfected samples with their corresponding non-transfected sample using a Student’s *t*-test.

Next, we measured the gene expression levels of *PER2* in HEPG-2 cells transfected with syn-miR-30c or anti-miR-30c, with or without TMAO incubation (Figure 5). TMAO decreased the levels of *PER2* mRNA after 8 and 24 h of incubation with TMAO. At 4 h, the inhibition of miR-30c-5p was accompanied by an increase in the expression of *PER2*, which was even higher in the presence of TMAO. At this timepoint, the overexpression of miR-30c-5p resulted in an increase in *PER2* expression, which was not exacerbated by the incubation with TMAO. The increase in *PER2* expression in the presence of TMAO and inh-miR-30c was also observed at 8, but not at 24 h. The overexpression of miR-30c-5p reduced *PER2* expression at 8 h, which TMAO did not increase. These results suggest that, in the absence of miR-30c-5p, TMAO could promote *PER2* expression (Figure 5).

## 3. Discussion

This study shows how TMAO, at a physiological dose (6 µM), modulates the expression of miRNAs related to lipid metabolism, atherosclerosis, and CVDs. We focused on miR-21-5p and miR-30c, as these were altered both in hepatocytes (HEPG-2) and macrophages (THP-1). Additionally, we validated our results in two non-immortalized cellular models, whose characteristics are likely to closely resemble what occurs in vivo, i.e., mice liver organoids and human primary macrophages. We found that miR-21-5p was upregulated in all models studied here (Figure 1 and Figure 2). In HEPG-2, miR-21-5p was significantly upregulated at 4 and 8 h of TMAO treatment and significantly downregulated at 24 h. The same trend was observed for mouse liver organoids, which showed a significant increase in miR-21-5p at 4 h of TMAO treatment and a significant decrease at 8 h, with no significant change at 24 h. In THP-1, miR-21-5p was upregulated at all timepoints. The same trend was observed in human primary macrophages. Therefore, our results show an early response of miR-21-5p to TMAO addition. miR-21-5p was shown to be involved in the resolution of acute inflammation [14,15,44]. This is consistent with an early upregulation by TMAO and a late decrease in its expression. In macrophages, both THP-1 cells and human primary macrophages, the inflammatory response could be more sustained because of their involvement in this process. This could explain the longer upregulation of miR-21-5p we observed in these cellular models [44]. miR-21 has been extensively studied in cancerous processes and has recently been linked to CVDs and inflammation [14,15]. It has been shown that circulating levels of miR-21 are increased in subjects with a higher risk of CVD death, with atherosclerosis [16], and with cardiogenic shock [45], suggesting its potential as a biomarker to assess CVD risk. Indeed, consumption of the Mediterranean diet with extra virgin olive oil seems to reduce circulating miR-21 [46]. It has also been observed that miR-21 plays a role in resolving inflammation and balancing pro- and anti-inflammatory responses [9]. Furthermore, it has been shown that miR-21 deficiency leads to foam-cell formation in vitro, demonstrating that it plays an essential role in atherosclerosis plaque progression and inflammation resolution [15]. Therefore, it seems that increased miR-21 expression lessens the inflammatory condition. Considering all the above, it could be suggested that TMAO may induce inflammation and cytokine release, thus affecting atherosclerosis progression, which could increase miR-21-5p expression aimed at counteracting the inflammatory state in both hepatocytes and macrophages.

miR-30c-5p was significantly upregulated in all biological models tested, especially at 8 and 24 h. This miRNA has been described to be involved in cholesterol and fatty-acid metabolism [12]. miR-30c regulates the assembly of apo-B-containing lipoproteins and reduces lipid biosynthesis and lipoprotein secretion [12]. As previously mentioned, TMAO was shown to alter cholesterol metabolism genes, [2,3]. Therefore, it could be suggested that the observed miR-30c-5p increase in all biological models and at all timepoints could be as a response to an alteration of cholesterol metabolism. Thus, miR-30c-5p could restore cholesterol metabolism genes by inhibiting their expression and, therefore, increasing its own expression. Indeed, it has been shown that mice fed a high-fat diet exhibited miR-30c overexpression and a reduction in hyperlipidemia and atherosclerosis due to decreased lipid synthesis and secretion of apo-B lipoproteins. Consistently, miR-30c-overexpressing Western diet-fed Apoe^−/−^ mice showed lower plasma cholesterol and triglycerides, as well as fewer and smaller atherosclerosis lesions [47]. In human subjects, miR-30c plasma levels were reduced in patients with altered lipid profiles and carotid plaques [46]. It has also been suggested that miR-30c could serve as a potential biomarker for CVDs and as a potential anti-atherosclerosis therapeutic target. We previously showed that miR-30c can be modulated by diet [39]. Hepatocytes and enterocytes supplemented with docosahexaenoic acid (DHA) showed increased miR-30c expression, confirming the link between this miRNA and lipid metabolism. Taking all this together, it could be suggested that TMAO may induce a reduction in APO-B-containing lipoproteins through the activation of miR-30c expression.

We found that both miRNAs target *PER2*. *PER2* significantly reduces its expression at 8 and 24 h, in accordance with miR-30c-5p and miR-21-5p upregulation. *PER2* and miR-21 have been linked to myocardial ischemia [48]. *PER2* is a central regulator of circadian rhythms and has been shown to elicit endogenous cardioprotection from ischemia [49]. miR-21, as discussed above, has been reported to play a cardioprotective role. In addition, it has been found that *Per2* overexpression, due to light exposure, was associated with a reduction in the infarct size related to myocardial ischemia in mice. It has also been described that mice lacking *Per2* had bigger infarct sizes and did not exhibit cardioprotection due to their limited ability to utilize carbohydrates [49]. Moreover, *Per2* knockout mice did not show appetite control and, when fed a high-fat diet, had more metabolic alterations related to obesity [50]. In human subjects, *PER2* has also been linked to metabolic syndrome. In fact, circadian rhythm disruption has been shown to be associated with a higher risk of CVDs. The Aragon Workers Health Study (AWHS) observed that those subjects with shift works that were more extreme (morning or evening versus central day shift), thus disrupting circadian rhythms the most, showed higher risk of atherosclerosis [51]. *PER2* expression in visceral fat was negatively correlated with waist circumference in morbidly obese subjects. Moreover, a single-nucleotide polymorphism in *PER2* has been linked to higher plasma cholesterol and triglycerides in subjects with metabolic syndrome [52]. All this evidence suggests that *PER2* disruption could lead to metabolic syndrome and altered plasma lipids, increasing CVD risk. Here, we demonstrated that TMAO exposure results in decreased PER2 expression at both mRNA and protein levels. In the presence of an anti-miR-30c, TMAO increased *PER2*. Therefore, our results suggest that TMAO may alter the circadian rhythm through *PER2*, and that this modulation of *PER2* could be mediated, in part, by miR-30c.

In conclusion, our results show that TMAO modulates the expression levels of miR-21-5p, a miRNA with a well-known role in inflammation, and of miR-30c-5p, involved in lipid metabolism. TMAO has previously been related to CVDs and atherosclerosis, and, according to our results, the impact of TMAO on these diseases could be mediated by its modulation of these microRNAs. Specifically, we found that TMAO upregulated these miRNAs and affected the expression of a common target gene, *PER2,* a negative regulator of the circadian system. 

## 4. Materials and Methods

### 4.1. Immortalized Cell Lines

HepG2 and THP-1 cells were obtained from the American Type Tissue Collection (Barcelona, Spain). Cells were maintained in RPMI for THP-1 and in DMEM for HepG2 (Lonza, Basel, Switzerland), with 10% fetal bovine serum (FBS), supplemented with 20 mM glutamine and a 1% mixture of antibiotics containing penicillin, streptomycin, and amphotericin B (Cultek, Madrid, Spain). THP-1 cells were differentiated into macrophages by incubating with 50 ng/mL phorbol 12-myristate-13-acetate (PMA) for 72 h (Sigma, Madrid, Spain).

### 4.2. Primary Human Macrophages

Peripheral blood mononuclear cells (PBMCs) were obtained from a young, healthy male donor. The study protocol was approved by the IMDEA Food Ethics Committee (PI-040, 05/03/2020), and the donor provided signed informed consent. PBMCs were isolated with Lymphoprep™ (StemCell Technologies, Grenoble, France) following the manufacturer’s instructions. PBMCs were maintained in RPMI with 10% fetal bovine serum (FBS) supplemented with 20 mM glutamine and a 1% mixture of antibiotics containing penicillin, streptomycin, and amphotericin B (Cultek, Madrid, Spain). Monocytes present in PBMCs were differentiated into macrophages by adding 15% culture medium from L-929 cells for 5 days.

### 4.3. Mouse Hepatic Organoids

All organoid reagents were purchased from STEMCELL technologies (Grenoble, France) unless indicated.

All procedures were carried out in accordance with the guidelines of the European Communities Directive 86/609/EEC. In addition, the protocols performed were approved by the Animal Ethics Committee (Proex 281/15 and Proex 282/15) of Ramón y Cajal Hospital (Madrid, Spain). Mice were housed in a standard animal facility and maintained under controlled conditions (25 ± 2 °C; 12 h light–dark cycle), with food and water available ad libitum. Male C57BL/6 mice were purchased from Charles River (Écully, France) and had a 7 day acclimatization period prior to use.

Mice were sacrificed by cervical dislocation. The liver was removed, dissected into 3–5 mm pieces, and incubated in a 37 °C water bath, with digestion solution (DMEM/F12 supplemented with 15 mM HEPES, dispase (1 U/mL), and collagenase IV (1 mg/mL)) for 20 min. After removing the tube from bath, liver pieces were vigorously pipetted up and down using a 10 mL serological pipette. Liver pieces were settled by gravity for 1 min, and the supernatant was collected and kept on ice. Fresh digestion solution was immediately added to the pellet for an additional 20 min of digestion, and this process was repeated until liver digestion was complete. The pooled digestion solution was passed through a 70 µm filter and subsequently through a reversible 37 µm strainer. The retained ductal fragments were then recovered by inverting the reversible filter and eluting with ice-cold Advanced DMEM/F12 (Thermofisher Scientific, Madrid, Spain). The resulting suspension was then pelleted at 290× *g* at 4 °C, for 5 min.

Matrigel (Corning, MA, USA) was added to the pelleted fragments and gently resuspended by pipetting. The suspension was pipetted into the center of the wells (20 µL/well) of a prewarmed 48-well plate. The plate was placed inside a 5% CO_2_ incubator at 37 °C for 15 min to allow the suspension to solidify in a dome shape. HepatiCult (350 µL/well) was then carefully added to submerge each dome, and the plate was placed back in the incubator at 37 °C. The medium was changed every 2–3 days, and organoids were passaged after 7 days. When a second passage was performed, organoids were first grown in HepatiCult for 3 days before the differentiation protocol was initiated (from this point onward, the medium was changed daily). On day 4 after passage, Hepaticult medium was changed to differentiation medium (Advanced DMEM/F12 supplemented with 1% penicillin/streptomycin, 1% GlutaMAX (Thermofisher Scientific, Madrid, Spain), 10 mM HEPES (Thermofisher Scientific, Madrid, Spain), 1:100 N2 supplement, 1:50 B27 supplement, 1 mM *N*-acetylcysteine, 10 μM DAPT, 50 nM A83-01, 10 nM recombinant human [Leu15]-gastrin I, 50 ng/mL recombinant mouse epidermal growth factor, and 100 ng/mL recombinant human fibroblast growth factor 10) as defined by Broutier et al. (2016) [53] for 9 days. For the concluding differentiation phase, medium was supplemented with 3 µM dexamethasone for 3 days.

### 4.4. Treatments of Cells with TMAO

A dose–response curve was performed in HEPG-2 and THP-1 cells with 6, 10, and 50 µM TMAO for 2, 4, 8, and 12 h and with 6 and 10 µM for 24, 48, and 72 h. We analyzed the expression of genes previously reported to be responsive to TMAO at all doses and timepoints in HEPG-2 and THP-1 cells. We analyzed the expression of ABCA1 and CYP7A1 in HEPG-2 cells and of ABCA1, CD36, and SR-A1 in THP-1 cells. We used 6 µM as the minimum dose, as this concentration has previously been measured in human blood. Tang et al. reported that median plasma TMAO levels, measured by liquid chromatography, were around 5.8 µM in heart failure patients [54] and increased to 8 µM after ingestion of two boiled eggs [10]. Koeh et al. reported that, after ingestion of an 8 oz steak, plasma TMAO levels increased up to 6 µM in omnivorous subjects at 24 h. Furthermore, fasted plasma TMAO levels ranged between 4 and 8 µM in these subjects [9]. As this study aimed to explore the effect of TMAO regarding dietary patters, the 6 µM dose was chosen as a physiological dose. We did not assay lower doses due to the fact that lower concentrations have not been measured in human blood. We assayed higher doses, 10 and 50 µM, to assess if there was a dose-dependent response and given the possibility that the minimum, physiological, dose could not be enough to modulate selected miRNAs. Based on these results, we selected 6 µM as the exposure dose for miRNAs analyses because it is a physiological concentration previously measured in human blood, and it effectively increased the expression of TMAO-responsive genes. We also selected specific exposure times for miRNA modifications in both cell lines. In HEPG-2, timepoints selected were 4, 8, and 24 h, whereas 12 and 24 h were selected for THP-1. Mice liver organoids were treated with 6 µM TMAO for 4, 8, and 24 h, and human primary macrophages were treated with 6 µM TMAO for 12 h.

### 4.5. miRNA-Enriched RNA Isolation

Cells were lysed with Tripure (Roche, Barcelona, Spain). RNA was isolated following the phenol–cloroform methodology adapted to enhance miRNA precipitation. Briefly, samples were incubated at −80 °C with Tripure for at least 1 h, and then 100 µL of chloroform was added per 500 µL of Tripure. Samples were centrifuged for 15 min at 4 °C, and the aqueous phase containing RNA was collected. RNA precipitation was achieved by incubation with isopropanol (1:1) for 16–20 h at −20 °C. Then, samples were centrifuged for 10 min at 4 °C, and the supernatant was discarded. The pellet was washed with 85% ethanol, centrifuged for 5 min at 4 °C, and finally resuspended in RNAse-free water.

RNA concentration was measured using a nanodrop 2000 (Thermofisher Scientific, Madrid, Spain), and RNA integrity was checked in 2% agarose gels.

MicroRNA-enriched RNA was isolated from human primary macrophages using the miRCURY RNA Isolation Kit-Cell and Plant (Exiqon Bionova, Madrid, Spain) following the manufacturer’s instructions.

### 4.6. miRNA Panel Selection

We applied a hypothesis-driven approach to select a panel of 22 CVD- or lipid metabolism-related miRNAs through web-based algorithms (Table S1). Briefly, miRNAs involved in atherosclerosis, cholesterol homeostasis, inflammation, CVDs, bile-acid synthesis, and lipoprotein metabolism were searched using Ingenuity Pathway Analysis (version 2015, Qiagen, Madrid, Spain), which provided a list of 29 candidates. miRNAs were then matched with genes involved in the pathways mentioned above and potentially modulated by TMAO using miRWalk 2.0 [55], based on miRbase 22.1. Twenty-two miRNAs were finally selected for further analyses.

### 4.7. Determination of Gene and miRNA Expression

Gene and miRNA expression levels were measured by real-time quantitative PCR (RT-qPCR). For gene expression quantification, total RNA was reverse-transcribed using the Prime Script Reverse Transcription kit (Takara, CA, USA), and the resulting cDNA was amplified using the FastStart Universal SYBR Green Master (Roche, Barcelona, Spain). Specific forward and reverse primers were used (Isogen Lifesciences, Barcelona, Spain). Gene expression levels were calculated using the 2^−ΔΔCt^ relative quantification method, comparing treated cells with each corresponding untreated control sample at each timepoint and normalizing with *RN18S* and *RPLP0*. miRNAs were reverse-transcribed using the miScript Reverse Transcription II Kit (Qiagen, Madrid, Spain) and amplified using the miScript SYBR Green PCR Kit (Qiagen, Madrid, Spain), using a specific forward primer (Isogen Lifescience, Barcelona, Spain) and a universal reverse primer included in the kit. miRNA levels were calculated using the 2^−ΔΔCt^ relative quantification method comparing treated cells with each corresponding untreated control sample at each timepoint and normalizing with *RNU6* and *RNU43*. Those miRNAs with Cts over 35 or over the non-template control were removed from the analysis.

### 4.8. Functional Analysis and Target Genes Selection

For those miRNAs significantly altered by TMAO, a functional analysis was performed to determine the KEGG pathways in which they are involved. To analyze the KEGG pathways of selected miRNAs, DIANA miRPath v0.3 was used [56], and a list of target genes was generated using different algorithms. First, we used MiRWalk [55] to obtain experimentally validated and predicted target genes by combining two different algorithms, TargetScan and miRDB, using stringent criteria (cutoff of energy ≤ −20 and 3′-UTR seed sequence location). Experimentally validated and predicted targets found by both algorithms were selected for further analysis [57,58]. A Venn diagram was constructed to check common target genes between selected miRNAs for further analysis using the Bioinformatics and Evolutionary Genomics online tool (http://bioinformatics.psb.ugent.be/webtools/Venn/ (accessed on 17 May 2021)). Functional GO biological processes and pathways annotated in genes targeted by both miR-30c-5p and miR-21-5p were identified using GeneCards version 5.5 (https://www.genecards.org/ (accessed on 18 May 2021); Weizmann Institute of Science).

### 4.9. Protein Quantification and Analysis

Cells were lysed in ice-cold NP-40 lysis buffer, and total protein was quantified using the Pierce TM BCA Protein assay kit (Thermofisher Scientific, Madrid, Spain). Equal amounts of protein were separated by SDS/PAGE, transferred onto nitrocellulose membranes, and probed with the indicated primary antibodies and the appropriate [59] HRP-conjugated secondary antibodies. Primary antibodies were used in the following concentrations: anti-Per2 (Abcam, Cambridge, UK) 1:500; anti CXCL16 1:2000; anti-IL12A 1:500 anti-Serpine1 (Biorbyt) 1:2000; anti-β-actin (Abcam, Cambridge, UK) 1:5000. Protein bands were visualized and measured with densitometry analyses using ImageJ software (version 1.8, Bethesda, Maryland, NIH, USA) [60]. Protein quantification was normalized with β-actin.

### 4.10. miRNA Inhibitors and Mimics Transfection

The miRNA-30c inhibitor (anti-miRNA-30c) and mimic (syn-miRNA-30c) were obtained from Dharmacon (Horizon Discovery, Waterbeach, UK). HEPG-2 cells were transfected with 50 nM anti-miRNA-30c or 5 nM syn-miRNA-30c using Oligofectamine (Invitrogen, Madrid, Spain) following the manufacturer’s instructions. All experiments included samples transfected with a negative inhibitor control (anti-miRNA-neg) or with syn-miRNA-1 as a positive control (Qiagen, Madrid, Spain). miRNA-30c inhibition or overexpression was confirmed by RT-qPCR as described above.

### 4.11. Statistical Analyses

All experiments were performed in triplicate, and biological triplicates were included in each experiment. Additionally, technical duplicates were included in all RT-qPCR reactions. Inconsistent replicates were repeated. We compared the expression levels of genes and miRNAs of each treatment timepoint with its corresponding untreated control sample using a Student’s *t*-test. For the analysis of the panel of miRNAs, we corrected miRNA expression levels for multiple testing with Benjamini–Hochberg FDR correction. Those miRNAs significantly altered at at least one timepoint with an FDR-adjusted *p*-value < 0.05 in both cell lines were identified as candidate TMAO-responsive miRNAs. miRNA expression levels of validation experiments and gene expression levels after TMAO exposure were compared at each timepoint with their corresponding control sample using a Student’s *t*-test, and raw *p*-values are shown. Statistical significance was set at *p* < 0.05. All analyses were performed with SPSS 19.0 (SPSS Inc., Chicago, IL, USA).

## Data Availability

Not applicable.

## Supplementary Materials

The following are available online at https://www.mdpi.com/article/10.3390/ijms222011145/s1.
