# Supplementary figures and images for: Trimethylamine n-Oxide (TMAO) Modulates the Expression of Cardiovascular Disease-Related microRNAs and Their Targets
Source: Int J Mol Sci. 2021 Oct 15;22(20):11145. doi: 10.3390/ijms222011145 (PMC8539082; doi:10.3390/ijms222011145)

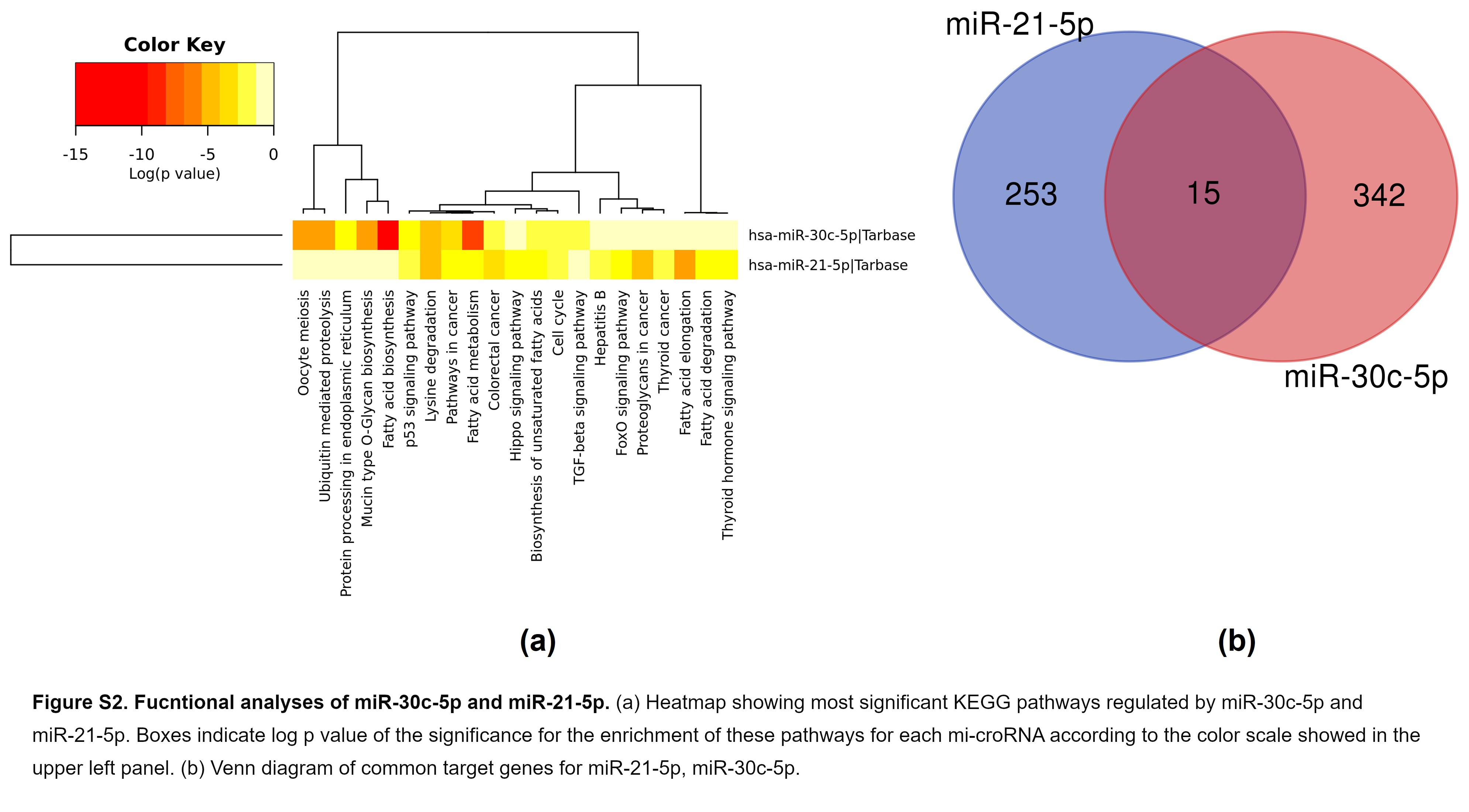

Supplement: Supplementary file 1 [file ijms-22-11145-s001.zip › Fig_S2_modified.jpg]

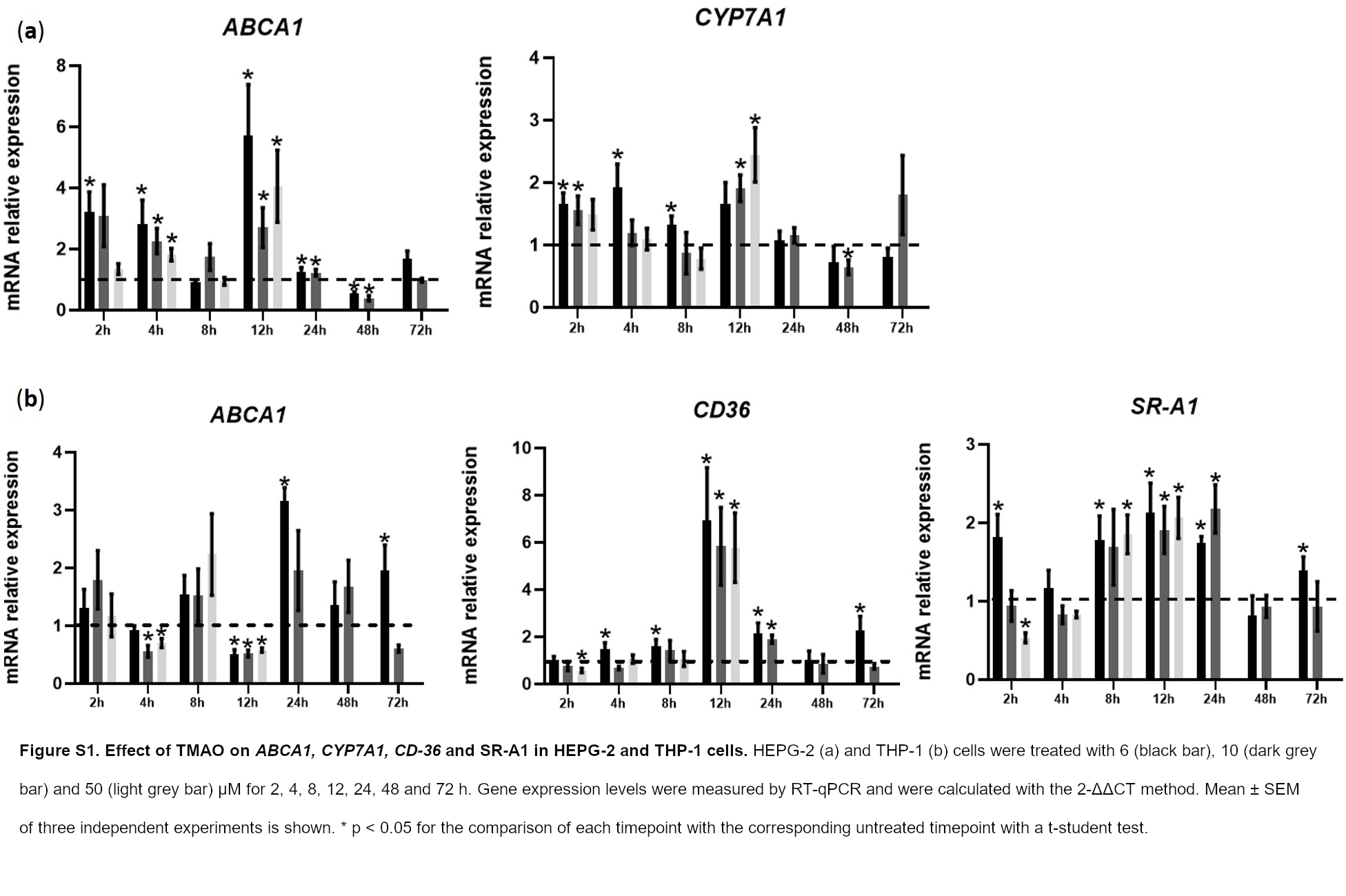

Supplement: Supplementary file 1 [file ijms-22-11145-s001.zip › Fig_S1_revised.jpg]
